# Exergaming and education: a relational model for games selection and evaluation

**DOI:** 10.3389/fpsyg.2023.1197403

**Published:** 2023-07-06

**Authors:** Daniel H. K. Chow, Stephen K. F. Mann

**Affiliations:** Department of Health and Physical Education, The Education University of Hong Kong, Hong Kong, Hong Kong SAR, China

**Keywords:** model of exergame, Bloom’s taxonomy, exergame selection, health and well-being, education

## Abstract

Exergaming, or technology-driven physical exercise, has gained popularity in recent years. Its applications include physical education, health promotion, and rehabilitation. Although studies have obtained promising results regarding the positive effects of exergaming, the outcomes of exergaming for different populations remain undetermined. Inconsistencies in the literature on this topic have multiple potential explanations, including the content and demand of the exergames and the capability of the exergamer. A model with a sound theoretical framework is required to facilitate matching between games and gamers. This article proposes a relational model based on a matrix of Bloom’s taxonomy of learning domains and the performance components of exergames. Appropriate matching of the physical demands of an exergame and the ability of the exergamer would enhance the effective usage of exergaming for individuals with various needs. This theory-based exergame model is developed to promote the general development, physical status, and psychosocial well-being of students, older adults, and individuals with rehabilitation needs. This model may provide a resource for future research on the application, effectiveness, and design of exergaming.

## Introduction

Exergaming has recently gained popularity among children and adolescents (Benzing and Schmidt, 2019; Milajerdi et al., 2021). A survey in Norway indicated that in 2020, 96% of boys and 76% of girls reported playing video games regularly, whereas the corresponding figures were 96 and 63% in 2018 (Norwegian Media Authority, 2020). A study in Canada involving 1,241 students in grades 10 and 11 revealed that 24% of the participants reported exergaming, and they played an average of 2 days per week for approximately 50 min each session (O'Loughlin et al., 2012).

Exergaming combines exercise and gaming and can also be called active video gaming. Advances in digital gaming technology have enabled a player’s body movements to replace the button presses of traditional sedentary gaming. Video gaming technology has advanced from motion-sensing handles to optical sensors that allow for more realistic whole-body movement. This development has prompted researchers to explore the potential applications of exergames and active video games in physical education (Annetta, 2008; Papastergiou, 2009; Staiano and Calvert, 2011; Perlman et al., 2012; Rüth and Kaspar, 2021), health promotion (Biddiss and Irwin, 2010; Emara et al., 2020), clinical rehabilitation (Anderson-Hanley et al., 2011; Chen et al., 2012; Howcroft et al., 2012; Vieira et al., 2021), and cognitive training (Green and Bavelier, 2012; Kiili and Perttula, 2012; Kolovelonis et al., 2023). Studies have demonstrated the potential beneficial effects of exergame interventions on physical rehabilitation for conditions such as Parkinson’s disease and stroke (Harris et al., 2015; Ribas et al., 2017; Cikajlo et al., 2020).

The present study defined exergaming, reviewed current research findings on its effects on different population groups, and recommended further research on its optimal use as an educational and physical exercise tool. We argued that exergaming is a beneficial tool that can (1) increase the level of physical exercise of the general public, (2) promote learning and physical education in students, and (3) facilitate rehabilitation. These purposes can be better achieved if the demands of exergames complement the player’s ability. Therefore, this study proposes a model with a multidimensional perspective to ensure precise matching of the demands of an exergame and the player’s ability.

## Scope of exergaming

### Promotion of exercise in children and adolescent gamers

One of the most widely recognized benefits of exergaming is its promotion of physical activity in children and adolescents (Daley, 2009; Biddiss and Irwin, 2010; Gao and Xiang, 2013). The growing global childhood obesity rate has attracted the attention of numerous experts. Insufficient physical activity is a primary cause of this trend (WHO Consultation on Obesity, 2000; World Health Organization, 2021). In contrast to traditional sedentary video games, exergames provide enjoyment and modest levels of physical exercise for players. Exergames offer physical benefits to children and foster sports-related interests and skills (Staiano and Calvert, 2011). Exergame players have a light to moderate level of energy expenditure, which is twice that of sedentary video game players (Lanningham-Foster et al., 2006; Maddison et al., 2007; Biddiss and Irwin, 2010; Ramírez-Granizo et al., 2020). In addition, different researches on children who participated in a graded exercise test indicated that high-intensity exergaming can increase heart rate and energy expenditure, which can affect vascular function and arterial adaptations in children (Mills et al., 2013; Chen and Sun, 2017). A randomized controlled trial revealed a small but definite effect of exergaming on body mass index and body composition in obese children (Maddison et al., 2011). These results indicate that exergaming is an adequate alternative to physical activity.

### Physical and psychological benefits of exergaming

Exergaming was demonstrated to improve children’s executive functioning (Kolovelonis et al., 2023). One study showed that an exergame involving repetitive physical activity similar to jogging on a treadmill enhanced children’s ability to resolve interference from conflicting visuospatial stimuli (Best, 2012). These effects were noted both in children and in older adults. A meta-analysis indicated that exergaming improved several functions in older adults, such as cognition, motor function, and balance (Fernandes et al., 2022). Another group of adult trainees who engaged in 24 sessions of 1-h exergame training had significantly greater improvements in their measures of physical function and their cognitive measures of executive control and processing speed than control participants did (Maillot et al., 2012).

Many exergamers experience enjoyment when playing. In addition, exergaming, as an alternative form of physical exercise, promotes psychological health. A study revealed that exergaming significantly improved reaction time, subjective happiness, and mental well-being in young adults (Hastürk and Munusturlar, 2022). Another study of 168 college students revealed that exergaming had short-term (approximately 10 min) but not immediate benefits compared to sedentary activity (Russell and Newton, 2008). In addition, exergaming promotes exercise self-efficacy. A study on the effects of group exergaming revealed that adolescents with obesity who participated in a dance-based exergaming group had significantly enhanced self-reported exercise competence and improved relationships with their parents (Wagener et al., 2012). One study indicated that exergaming helped alleviate depression (Li et al., 2016).

Traditional video games also have significant positive effects on multiple health-related outcomes. One review indicated that sedentary video games improved 69% of psychotherapy outcomes, 59% of physical therapy outcomes, 50% of physical activity outcomes, 42% of health education outcomes, 42% of pain control outcomes, and 37% of disease self-management outcomes (Primack et al., 2012). Although no study has analyzed the effects of exergaming on these outcomes, similar effects can be reasonably predicted.

## Pitfalls of exergaming

Although considerable evidence indicates that exergames enhance physical and psychosocial health in children, some researchers hold opposite opinions. A naturalistic study investigated whether children playing a new exergame spontaneously engaged more in physical activity than those playing a sedentary game; the evidence did not indicate that children playing active video games were more active in general (Baranowski et al., 2012). This result also raises the question of whether exergaming can affect the long-term physical health of children.

A review of 14 descriptive studies, one uncontrolled trial, and two pilot randomized controlled trials concluded that evidence remains mixed on the subject. No adequately powered randomized controlled trial has assessed the long-term effects of exergaming on children’s health (Daley, 2009).

Rüth and Kaspar (2021) described multiple potential critical pitfalls of exergaming, which included the following: (1) unhealthy exercise due to inappropriate training intensity; (2) negative motivational, emotional, and cognitive effects of the competitive mode that hamper physical activity or cause undesirable side effects; and (3) experiences that are not shared or considered worthy of discussion. When exergaming is used as a form of physical education, education- and health-related topics are not discussed during the exergame. Opportunities for communication are limited, and discussion may result in conflicts with others.

These drawbacks may not result from exergaming itself but from the selection of exergames. The content and difficulty level of a game may result in improper matching with the gamer’s ability, which may affect the usage of exergaming.

## Choice of exergame

Exergames are interactive and provide physical exercise and workouts rather than promoting the behaviors (e.g., fighting) of traditional sedentary video games. Thus, whether exergames also produce the negative effects of conventional video games remains unclear. Although studies have reported the negative effects of video games, no studies are known to have reported the adverse effects of exergaming.

Some authors have contended that employing video games as a learning mode can be more enjoyable and stimulating than traditional modes of teaching and learning (Prensky, 2001; Gee, 2003; Kiili and Perttula, 2012). Teachers have employed video games for textbook replacements, homework assignments, and virtual learning (Annetta, 2008). Exergaming is an appealing educational tool for children and adolescents. Nevertheless, because of the mixed results on the effects of exergaming, additional research should investigate factors such as players’ preferences and the difficulty levels of games (Daley, 2009).

Empirical research on why and how games affect students is essential as exergaming gains attention and popularity in education. Therefore, the present study constructs a classification system for exergames to facilitate future research and to effectively incorporate exergaming into educational contexts.

### Classification of exergames

Studies have classified video games by function, form, and genre (Solomon, 1984; Rebetez and Betrancourt, 2007; Sheehan and Katz, 2010). Solomon (1984) proposed a classification of video games that includes (1) simulations (the game reflects reality), (2) abstract games (the game itself is the focus of interest), and (3) sports. Another study classified video games into six types, namely (1) general entertainment, (2) educational, (3) fantasy violence, (4) human violence, (5) nonviolent sports, and (6) sports violence (Funk and Buchman, 1996).

The Exergame Network, an international collaboration of health and fitness practitioners, exergame developers, and researchers, proposed the following five basic types of exergames: (1) workout exergames—the player completes a workout based on the advice of the game; (2) control exergames—the player uses different body parts to control the game; (3) exergame machines—the player uses real fitness equipment to play the game; (4) sensory exergames—the player must jump and run to earn points; and (5) rhythm exergames—the player becomes a musician or dancer and is guided by music (The Exergame Network, 2012). This system classifies exergames by their motion and perception demands on the player and may be inadequate for enabling exergame adoption in children’s education.

Exergames must be individualized and tailored to the target population to yield the greatest benefit (Mishra et al., 2016). Because individualized exergame designs are complex and costly, developing a comprehensive model to facilitate the tailoring of exergames is essential.

### Components of exergames from a learning perspective

A growing number of scholars have recommended using exergame learning applications, particularly in physical education (Fogel et al., 2010; Staiano and Calvert, 2011; Best, 2012; Kiili and Perttula, 2012; Ramírez-Granizo et al., 2020). Learning objectives are required when exergames are used as a learning and teaching tool. They are the building blocks of conceptual knowledge and the skills that help students develop understanding and strength.

Bloom (1956) proposed a taxonomy that classifies learning into three separate domains, namely the (1) cognitive domain, (2) affective domain, and (3) motor domain (Forehand, 2005; Huitt, 2011; Hoque, 2016). It is a model to categorize and organize different levels of learning. Cognitive domain includes intellectual skills and abilities related to knowledge, understanding, and thinking. It is further divided into six levels, which are remembering, understanding, applying, analyzing, evaluating, and creating (Huitt, 2011). Affective domain, on the other hand, includes emotional and social skills and abilities related to attitudes, values, and beliefs. It focuses on how students develop feelings, interests, and attitudes toward learning, and how they interact with others. The affective domain is divided into five levels, which are receiving, responding, valuing, organizing, and characterizing (Savickiene, 2010). About the psychomotor domain, it includes physical skills and abilities related to movement, coordination, and dexterity. The psychomotor domain is less commonly used than the cognitive and affective domains but is relevant for subjects that require hands-on skills or physical activity. The psychomotor domain is divided into six levels, which are perception, set, guided response, mechanism, complex overt response, and adaptation (Begam and Tholappan, 2018).

By including all three domains in education planning, it is able to create a holistic approach to learning that addresses not only the intellectual but also the emotional and physical aspects of student development. This can lead to a more comprehensive and effective educational experience for learners (Krathwohl, 2002; Halawi et al., 2009; Seaman, 2011).

The present study employs Bloom’s taxonomy as a framework to analyze the effects of exergaming on exergamers (Cooper and Higgins, 2015). The primary purpose of broadening exergame use in learning is to enhance motor, cognitive, and emotional–social skills. A multidimensional perspective should be employed to analyze the potential uses of exergaming. Precise matching between the demands of an exergame and the ability of the exergamer is crucial.

**Figure 1:**
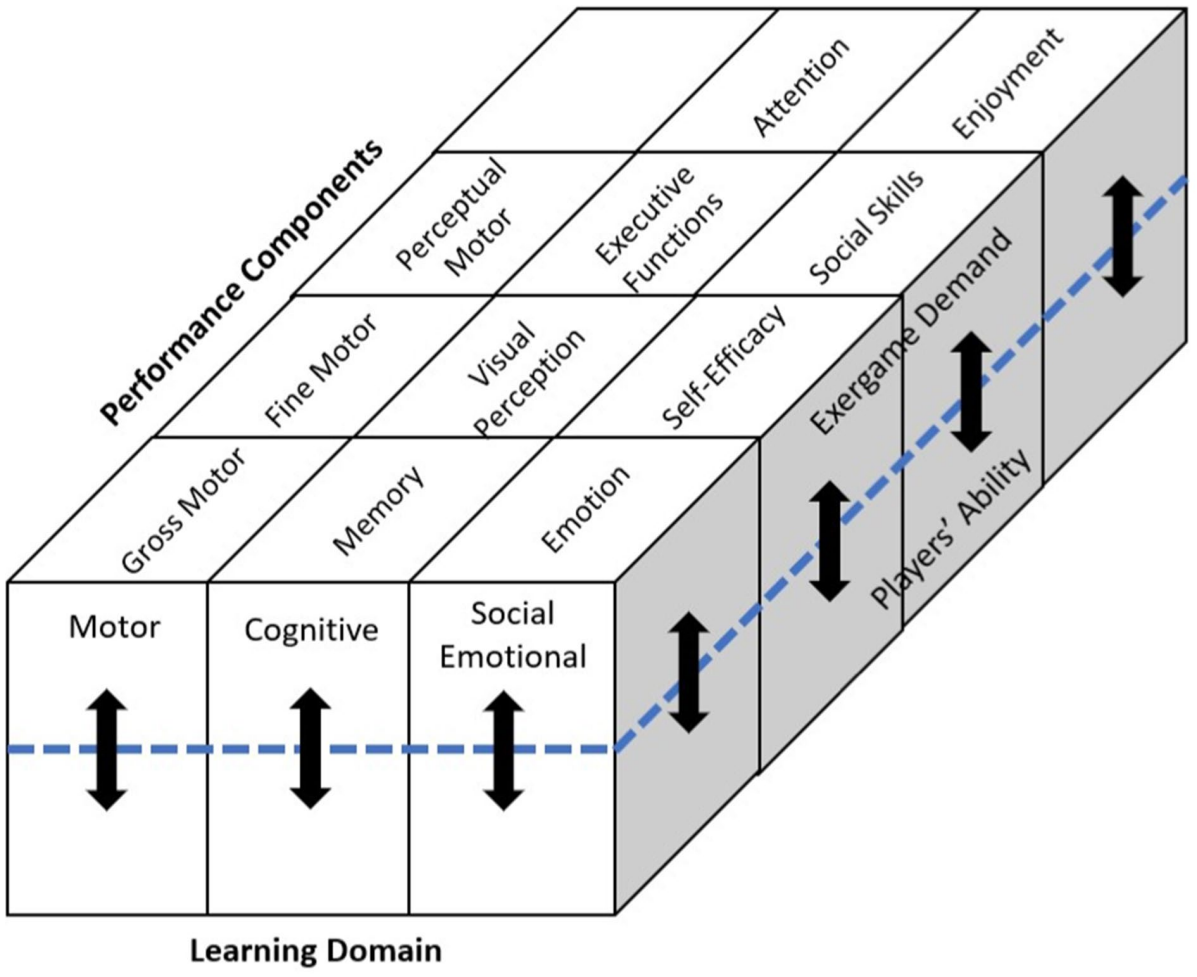
Matrix of learning domains, performance components, and matching between demands of exergame and ability of exergamer.

Subdivision of the learning domains can provide a detailed framework for the analysis of exergame demands (Figure 1).

The ability to control voluntary muscle movement is critical to motor development. Motor skills are generally divided into gross and fine skills; gross motor skills involve the use of large muscles in the arms, legs, and torso, and fine motor skills involve the precision, dexterity, and coordination of the hands and fingers (Logan et al., 2018). The motor domain of this model comprises three basic performance components: (1) gross motor skills, (2) fine motor skills, and (3) perceptual motor skills (Bayley, 1999; Frost et al., 2012). These abilities encompass the fundamental motion and coordination demands of exergaming and are also key aspects of physical development. Gross motor skills include balance and coordination, posture control, and whole-body movement, skills that are commonly required in many exergames (Reynolds et al., 2014).

Cognitive development, evidenced by one’s ability to think and understand, results from information processing, concept formation, perceptual skills, language learning, and other brain functions (Richardson, 2019). The cognitive domain in our proposed model is divided into the following performance components: (1) memory, (2) visual perception, (3) executive functions, and (4) attention (Greeno et al., 1996). It is unapparent that most exergames demand several cognitive skills. All exergames require a certain level of attention and visual perception. In addition, memory and executive functions are closely associated with the player’s response to an exergame (Eggenberger et al., 2020; Zhao et al., 2022).

Social development reflects a child’s ability to interact with peers and adults in a socially acceptable manner. Video gamers are not confined to an isolated console but are connected to many other gamers worldwide through the Internet (Kooiman and Sheehan, 2015). This technological advancement imbues exergames with social and emotional elements (Li et al., 2018). The social–emotional domain of the proposed model is divided into the following performance components: (1) emotion, (2) self-efficacy, (3) social skills, and (4) enjoyment (Harris, 1988; Moissinac, 2003). These factors are crucial in the social development of children (Han and Kemple, 2006).

### Matching exergame demand and player ability

More vigorous research studies are required because of the growing interest in exergames as educational and rehabilitation tools. Although adverse effects of exergames have not been reported, exercising caution is crucial in the developmental stage. Notably, when adopting exergaming as a teaching and learning tool, the demands of the exergame should be carefully matched with the player’s motor, cognitive, and social abilities. The present study’s proposed classification system may inform pedagogical practices and provide insight into the integration of exergaming and education. In addition, this model may provide an essential reference for future exergaming regulations prior to its inclusion in education curricula.

Undeniable differences are present between real sports and exergaming. Replacing real sports with exergaming for children requires careful contemplation. The proposed classification system may guide the systematic study of the effects of exergaming and the dissimilarities between real sports and the sport-like activity of exergaming. Many advancements may be made in exergaming hardware and software, and systematic studies of the effects of exergaming should guide such advancements.

The proposed system may contribute to the subsequent development of a standard-level classification system, which can ensure the positive and healthy growth of exergaming. Until now, the adverse effects of exergaming remained unexplored; this study discussed both the positive and negative effects of exergaming on the health and well-being of children. Relevant studies have revealed a trend in the increased use of exergames in the educational context.

## Conclusion

This article summarizes the benefits of exergaming on gamers’ physical and psychosocial well-being. Although the potential benefits of exergaming have been reported, selecting suitable exergames to match the needs of gamers is crucial to optimize these benefits. This study proposed a model based on a matrix of learning domains, the performance components of exergames, and the matching of the exergame demands with the ability of the exergamer. Based on Bloom’s taxonomy (Bloom, 1956) of learning domains, the model enables the proper and effective selection of exergames for various purposes.

An exergame analysis is essential to maximize the benefits of exergaming with respect to rehabilitation and training. A wide variety of highly individualized exergames are commercially available and include multiple therapeutic elements. This study adopted a learning perspective framework to analyze the contents of exergames for multiple reasons. First, exergames have been widely employed to enhance children’s physical, cognitive, and mental functioning. An analysis based on a learning perspective is therefore appropriate. Second, for different populations, exergaming can be considered a growth process through which the gamer acquires skills to enhance their overall well-being. Thus, a developmental evaluation would be most appropriate for assessing exergame types.

Finally, the appropriate matching of gamers and exergames may determine the efficacy and effectiveness of exergame training. Some contradictory findings regarding the effectiveness of exergaming are believed to have resulted from inappropriate matching between the needs of gamers and the demands of exergames. Therefore, the proposed theoretical framework for the use of exergames can help maximize the benefits of exergaming.

## Author contributions

DC contributed to the conceptual design and model development. SM contributed to the model development and preparation of the manuscript. All authors contributed to the article and approved the submitted version.

## Conflict of interest

The authors declare that the research was conducted in the absence of any commercial or financial relationships that could be construed as a potential conflict of interest.

## Publisher’s note

All claims expressed in this article are solely those of the authors and do not necessarily represent those of their affiliated organizations, or those of the publisher, the editors and the reviewers. Any product that may be evaluated in this article, or claim that may be made by its manufacturer, is not guaranteed or endorsed by the publisher.
